# The impact of subsidized low aromatic fuel (LAF) on petrol (gasoline) sniffing in remote Australian indigenous communities

**DOI:** 10.1186/s13011-017-0121-6

**Published:** 2017-08-17

**Authors:** Peter d’Abbs, Gillian Shaw, Emma Field

**Affiliations:** 1 Menzies School of Health Research, Brisbane, Australia; 2 School of Public Health, University of Queensland, Brisbane, Australia; 3 Nous Group, ACT, Canberra, Australia

**Keywords:** Petrol sniffing, Gasoline sniffing, Inhalants, Indigenous health, Supply reduction, Low aromatic fuel

## Abstract

**Background:**

Since 2005, the Australian Government has subsidized the production and distribution of Low Aromatic Fuel (LAF) as a deterrent against petrol (gasoline) sniffing in remote Indigenous communities. LAF is used in place of unleaded petrol as a fuel for vehicles and other engines. This paper reports findings from an independent evaluation of the LAF rollout.

**Methods:**

Forty one Indigenous communities were surveyed between 2010 and 2014, with each community being visited twice at a two yearly interval. Quantitative data on prevalence of petrol sniffing were collected, as well as qualitative data on the acceptability of LAF, evidence of substitution for inhaled petrol with other drugs, and programs such as recreational, training and employment opportunities. Prevalence rates of sniffing per 1000 population for each survey year and community were calculated by dividing the total number of sniffers by the population aged 5–39 years and multiplying by 1000.

**Results:**

Between 2011–12 and 2013–14, the total estimated number of people sniffing petrol declined from 289 to 204, a fall of 29.4%. At both times, the median petrol sniffing prevalence rate was lower in communities with LAF than in communities without LAF. In 17 of the 41 communities, comparable data were available over a longer period, commencing in 2005–06. Fifteen of these communities stocked LAF over the entire period. In these communities, the median rate of petrol sniffing declined by 96%, from 141.6 per 1000 population in 2005–06 to 5.5 in 2013–14 (*p* < 0.05). LAF was widely accepted, although acceptance was often qualified by a belief that LAF harmed engines. Anecdotal reports suggest that the fall in petrol sniffing may have been offset by increased use of cannabis and other drugs, but the relationship is not one of simple cause-and-effect, with evidence that an increase in cannabis use in communities commenced before the LAF rollout began. Provision of services in communities has improved in recent years, but many programs continue to be inadequately resourced.

**Conclusions:**

The rollout of LAF appears to have contributed to reducing petrol sniffing and associated harms in Australian Indigenous communities.

## Background

This paper presents findings from an evaluation of the impact of introducing government-subsidized Low Aromatic Fuel (LAF) as a deterrent to petrol sniffing (also known as gasoline sniffing) in a national sample of 41 Indigenous communities in Australia, most of them in remote regions.

Volatile substance misuse (VSM) has been described as ‘an under-recognized and underestimated global public health issue’ [1], particularly among young people in marginalized populations. As a form of recreational drug use, VSM refers to the use of chemical compounds – sometimes referred to collectively as inhalants - that emit fumes at room temperature. There are literally hundreds of inhalants in everyday use and readily available. They are usually grouped into four categories: volatile solvents (eg solvent-based contact adhesives, petrol); aerosols (e.g. spray paints, deodorants); gases (eg butane lighters, propane tanks) and nitrites (eg isoamyl nitrite) [2]. Petrol sniffing is a form of VSM that has long had particularly damaging consequences among young people in Indigenous communities in several countries, including Australia, New Zealand, the USA and Canada [3, 4]. For example, in a review of 16 youth suicides in the Pikangikum First Nation between 2006 and 2008, the Office of the Chief Coroner of Ontario, Canada, reported that almost all of the youths involved were inhalant users, and that young girls in grades 3 and 4 had recently self-reported that up to 27% had tried sniffing petrol [5].

Prevalence of VSM tends to be under-estimated as a result of the young age at which it often commences, and the marginal status of many users, both of which can lead to their being omitted from the school settings in which youth drug use surveys typically occur. It occurs in developing countries such as Mexico and India, where it has been found to be more widespread among children and adolescents living in the street than with families [6–10], in societies in transition such as Bulgaria and Russia, and in what Gigenjack has called ‘pockets of poverty and deprivation’ in wealthy countries such as Australia, Canada and UK [8]. Baydala [11] suggests that the higher prevalence found in indigenous populations may be a result of socio-economic disadvantage rather than ethnic attributes. Halliburton and Bray examined trends in inhalant use among 8th, 10th and 12th grade students in US schools between 1991 and 2011 and found a decline in use among both males and females, but a rising proportion of females among users. The mean levels of lifetime use over the period were 17.6% for eighth grade students, 15.2% for tenth grade, and 13.4% for twelfth grade students [12]. In Europe, reported rates range between 18% of students in Ireland and 3% in Bulgaria [9]. The United Nations’ *World Health Report 2003* reported that, among 40 countries for which lifetime inhalant usage data was available for the 1990s, 10 countries reported rates of 10% to 20% of the youth populations examined, 15 reported rates between 5% and 10%, and 16 reported rates below 5% [4].

In Australia, the two most widespread forms of VSM are petrol sniffing among Indigenous youths, particularly in remote communities, and ‘chroming’ – or inhalation of aerosol paints – in urban settings by both Indigenous and non-Indigenous youths. Prevalence data are scarce and often unreliable, in part because levels of VSM in any one place can fluctuate wildly, and in part because many inhalant users are under the minimum age at which drug use surveys conventionally sample populations. The largest school-based survey in Australia is a three-yearly survey of secondary students’ use of alcohol, tobacco, over-the-counter and illicit substances conducted by Cancer Council Victoria. The 2014 survey, involving a sample of 23,007 students aged 12–17 years drawn from 352 schools, found that inhalants were the most commonly used illicit substances among younger students, and second only to cannabis among older students. In contrast with cannabis, alcohol and other substances, inhalant use declined with increasing age. Among 12–15 year old students, 17.8% reported having ever used inhalants, with 7.0% having done so in the past month. In comparison, 10.1% of 12–15 year old students had ever used cannabis, 4.5% in the past month. Among 16–17 year old students, however, the picture was reversed: only 3.9% reported having used inhalants in the past month, compared with 12.7% reporting using cannabis [13].

Inhalants generate a rapid ‘high’, sometimes accompanied by hallucinations. They may be used primarily for the pleasures of the resulting ‘buzz’ or, in some settings, to stave off hunger and coldness, or simply as an antidote to boredom [4, 14]. As Medina-Mora and Real note, the toxic effects of inhalants may be stronger among the children and adolescents who use them than among adults [9]. VSM has been associated with a number of serious medical and psychosocial conditions including ‘sudden sniffing death’ from ventricular arrhythmia [15] and death from asphyxiation as a result of petrol displacing oxygen from the lungs [16]. It can lead to a range of neurological and cognitive impairments, including learning and memory impairment, as well as damage to kidneys, liver, heart and lungs [15]. Prenatal exposure to volatile substances has been associated with low birthweights, prematurity, spontaneous abortion and neurobehavioural problems [3]. In addition to harms experienced by inhalant users themselves, VSM can have a broader impact on families and communities, through behaviours such as interpersonal violence and vandalism inflicted by intoxicated users [17].

In comparison with inhalant users in urban areas, petrol sniffing in remote communities in Australia has often commenced at a younger age and persisted for a longer period, leading to higher levels of chronic use, with the associated harms [3, 17]. Estimating VSM-related mortality and morbidity is hampered by an absence of systematic data collection, with deaths and illnesses often attributed to immediate causes, such as burns or injuries, that in reality have been precipitated by VSM. On the basis of coronial and other evidence, it has been estimated that, between 1981 and 1991, 63 Indigenous Australians died from causes linked to petrol sniffing, with another 37 dying from similar causes between 1998 and 2003 [3].

Interventions for petrol sniffing and other forms of VSM have long been hampered by lack of evidence of intervention options and outcomes. A systematic review of interventions for inhalant dependence, conducted in 2010 and restricted to randomised-controlled trials and controlled clinical trials, was unable to identify any relevant studies [18]. In 2012, MacLean et al. conducted a second systematic review, broadening their inclusion criteria to include comparative studies with or without current controls and case series with post-test or pre-test/post-test outcomes [19]. They found 19 studies that met their inclusion criteria, covering one or more of case management, counselling, recreation and engagement activities, and residential treatment, but were unable to identify any firm conclusions to support evidence-based therapeutic approaches to VSM.

The use of LAF as a deterrent to petrol sniffing in Indigenous communities commenced in 2005 and grew out of a series of community-based supply reduction initiatives that demonstrated both positive outcomes and strong community support [20–22]. The origins and evolution of these initiatives have been described elsewhere [23]. The first and best known LAF was developed by BP Australia and marketed as Opal fuel, as a 91 Octane fuel suitable for use in all engines designed for 91 Octane regular unleaded petrol (RULP). More recently, Viva Energy Australia (formerly Shell Company of Australia) has also begun producing LAF. Because LAF contains much lower levels of aromatics than RULP, inhaling it does not lead to intoxication (although it can still be harmful if inhaled since, like other volatile substances, it can displace oxygen from the lungs).^1^ Since 2005, the rollout of LAF has been facilitated by a Commonwealth Government subsidy of 33 cents per litre, paid to BP to offset the higher production costs of the fuel and to enable the fuel to be retailed at a price competitive with RULP. The scheme has proved highly popular. By July 2012, a total of 123 sites throughout regional and remote Australia stocked LAF, including 74 Indigenous communities, 40 service stations or roadhouses, and nine ‘other’ outlets. Funding for the rollout was $115.9 million over the five years commencing 2011–12 [24]. By 2016, the total number of LAF outlets had risen to 175.^2^

The rollout of LAF has been monitored through several independent evaluations.

In 2005, two of the authors of this paper were engaged by the then Commonwealth Department of Health and Ageing to collect baseline data on prevalence of petrol sniffing in 88 communities that were either using or eligible to use LAF, and to devise a data collection method and instruments suitable for ongoing monitoring. In 2008, 19 of these communities were re-visited as part of an evaluation of the impact of LAF. The study found that in 17 of these the prevalence of petrol sniffing had declined, and that across the whole sample there had been a decrease of 70% in the number of people sniffing petrol. Qualitative findings indicated that, in communities experiencing a decline in sniffing, most people attributed the change wholly or partly to the introduction of Opal fuel.^3^ In 2010, we were again commissioned by the Department to conduct a broader examination of the impact of LAF, its acceptability in communities, and the presence and adequacy of other factors shaping community-level responses to petrol sniffing. Fieldwork for this study was conducted between 2011 and 2014. This paper presents key findings from the most recent study. Further details are contained in a final report of the study [25].

## Methods

The 2010–2014 study was conducted in a sample of 41 Indigenous communities, selected by the Department of Health and Ageing in consultation with ourselves, initially on the basis of three criteria: the availability of baseline data against which to monitor the impact of LAF; a need to represent all regions in which LAF had been introduced, and agreement by the community to participate in the study. A fourth criterion was subsequently added by including several communities where petrol sniffing was reported to be an emerging problem. Each community was visited twice, at two-yearly intervals, in the course of the study, in 2011–12 and again in 2013–14.

The study had three objectives: first, to estimate the prevalence of petrol sniffing; second, to elicit qualitative feedback on community perceptions about the impact of LAF on sniffing and its acceptability for use in vehicles, and third, to assess and document aspects of service provision which might be expected to have an impact on sniffing – for example access to youth programs.

Quantitative data on petrol sniffing prevalence was obtained using a variant of a ‘proxy respondent’ procedure pioneered in the 1990s by an Aboriginal community-controlled health service - Nganampa Health - in the Anangu Pitjantjatjara Yankunytjatjara (APY) Lands of South Australia [26–28]. A data collector would sit down with a population list of people aged 10–39 in each community, and read out each name on the list with informants such as Aboriginal Health Workers. The informant would identify which people sniffed, and how often they sniffed. The process was repeated with three informants in each community, and results collated to produce a table showing numbers and age/sex categories of people who sniffed petrol, and frequencies of sniffing.

The proxy respondent procedure was chosen over self-report surveys because, while the latter have been used successfully in studies of single communities (eg [29]), it would be prohibitively resource-intensive in a multi-community study such as this, particularly given that petrol sniffing is usually a clandestine activity, conducted at night by young people who are often not readily accessible during the day.

However, not all communities could make accurate population lists available, and in the larger communities that did so, the lists were too long to reasonably ask informants to peruse every name. For this study, therefore, we adopted a modified procedure, in which fieldworkers began with a series of categories, chosen not only to create mutually exclusive fields, but also to accord with the ways in which people on the ground in communities thought about people’s ages. The categories used were as follows:

- Primary school aged girls
- Primary school aged boys
- Young women - high school, too young to go to pub
- Young fellas – high school, too young to go to pub
- Older women – people who can buy grog (a widely used slang term for alcoholic beverages)
- Older men – who can buy grog.

A fieldworker would first ask ‘Can you think of any little girls – primary school kids – who sniff? If a person was identified, their initials only were recorded. For any person identified as sniffing, the fieldworker would then ask the informant to identify the person’s age and sniffing frequency – using a frequency matrix described below. The fieldworker would also record the first names and initials of identified users in order to compare the list of persons identified with those identified by other informants. In both the original and modified procedures, data collectors were instructed that if two or more people identified a person as someone who sniffed petrol, then that identification was considered valid. If only one person identified someone, but they were considered to be in a good position to know – for example a family member – then that identification was also accepted as valid. In the event of conflicting assessments of the same person’s petrol sniffing status, fieldworkers were instructed to use their judgement as to the most knowledgeable informants. The same rules applied for establishing the frequency with which people sniffed.

Once numbers of people sniffing had been collated, the data collector aggregated the numbers in each age x gender x frequency category, and entered the aggregates into a table. This was the data taken from the community; sheets with first names and initials were not taken from the community.

Sniffing frequency categories used were the same as those used in the earlier studies of the LAF rollout, and were in turn adapted from those developed by Nganampa Health in their earlier studies. The categories and associated definitions are shown in Table 1. As the Table indicates, the basis for categorizing a person is their sniffing-related behaviour over the six months prior to data collection.

**Table 1:** Definitions of sniffing frequency categories

Category		Definition
Non-sniffer		Not known to have sniffed petrol or any other inhalant in past 6 months.
Current sniffer	Experimental/occasional	Believed to have sniffed petrol or other inhalant in past 6 months, but no evidence of regular use.
	Regular	Believed to have sniffed petrol or other inhalant regularly over past 6 months, but does not meet criterion of heavy use (i.e. at least once a week).
	Heavy	Has sniffed petrol or other inhalants at least weekly (whenever inhalants are available), over past 6 months.

Qualitative data was collected by fieldworkers’ observations and interviews. Topics covered included:

- Community views regarding impact of LAF in the community;
- Anecdotal reports of use of other substances such as marijuana (and any information on individuals who have changed their habits from sniffing petrol to smoking marijuana or other drug use);
- Nature and accessibility of youth, recreation, and alcohol and other drug services;
- Nature and use of employment and training services.

After each of the two data collection rounds, results relating to individual communities were relayed back to these communities in a plain language format.

In 17 of the 41 sample communities, comparable data on prevalence of petrol sniffing is available over a longer period, at two additional time points, namely 2005–06 and 2007–08. These data are incorporated in the analysis below. Prevalence rates of sniffers per 1000 population for each survey year were calculated by dividing the total number of sniffers by the population aged 5–39 years and multiplying by 1000 for each community. Estimated Resident Populations based on the Australian Bureau of Statistics Census of Population and Housing in 2006 and 2011 were used, the former for calculating the prevalence rates in 2006 and 2008 and the latter for calculating prevalence rates in 2012 and 2014. The median prevalence rates of communities with LAF and communities without LAF where calculated. The Wilcoxon rank-sum test was used to test the difference between median prevalence rates for communities with and without LAF and the difference in median prevalence rate between years.

In order to examine the relationship between declining petrol sniffing prevalence and reported use of other drugs, levels of concern with alcohol and cannabis in the 18 communities that recorded a decline in petrol sniffing between 2011–12 and 2013 – 14 were compared with levels of concern in those communities that recorded no decline. A similar comparison was made for reported use of inhalants other than petrol. Fishers exact test was used to measure the association between a decrease in prevalence of sniffers and reports of heavy use and/or major concerns with cannabis or alcohol use. Analyses were performed in Stata 14.2.

## Results

In the 41 communities surveyed, the total estimated number of people currently sniffing petrol declined from 289 in 2011–12 to 204 in 2013–14, a decline of 29.4%. The decline was not uniform across communities or regions, with 18 communities recording a decline, 15 communities an increase in numbers of current sniffers. In the eight remaining communities, no people were reported as sniffing in either 2011–12 or 2013–14.. Most current sniffers at both data collection periods (79.3% in 2011–12 and 77.5% in 2013–14) were male. As Table 2 shows, a little over half of current sniffers at both periods were aged 15–24. However, as the Table also shows, the number of older sniffers fell both absolutely and as a proportion of the total, while the number of very young sniffers – aged 5–9 – increased from 4 to 8. Any reported petrol sniffing amongst children of this age is obviously a cause for serious concern and continued monitoring, especially evidence of an increase. However, given the very small numbers involved, caution in interpreting the figures is appropriate.

**Table 2:** Trends in numbers of current sniffers, 2011–12 and 2013 – 14 (40 communities)*

Current sniffers	2011–12		2013–14		% change
	No.	%	No.	%	
Age-group					
5–9	4	1.5	8	4.1	100.0
10–14	79	29.7	69	35.4	− 12.7
15–24	146	54.9	102	52.3	− 30.1
25–39	37	13.9	16	8.2	− 56.8
Total	266	100.0	195	100.0	− 26.7
Frequency					
Occasional	142	53.4	106	54.4	− 25.4
Regular	51	19.2	54	27.7	5.9
Heavy	73	27.4	35	17.9	− 52.1
Total	266	100.0	195	100.0	− 26.7

*Age and frequency breakdowns were missing for one community with 23 current sniffers in 2011–12; this community has been excluded from the table, hence the smaller totals

The decline in the total numbers of current sniffers, as Table 2 shows, was accounted for by falls in the numbers of occasional sniffers and heavy sniffers, but not regular sniffers.

**Fig. 1:**
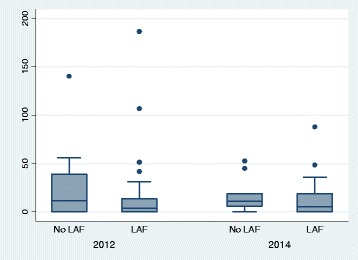
Prevalence of petrol-sniffing per 1000 population by LAF availability, 2012 and 2014 (41 communities)

Of the 41 communities, 28 reported availability of LAF for both 2011–12 and 2013–14; eight had no LAF for both years, three reported no LAF in 2011–12 and LAF in 2013–14, and two reported LAF in 2012 but no LAF in 2013–14. In 2012, communities with LAF had a lower median petrol sniffing prevalence rate (3.9) than those without LAF (11.2). The difference, however, was not statistically significant (*p* = 0.51) largely because of the small frequencies and large inter-quartile ranges involved (see Table 3) and the presence of several outliers, especially in communities with LAF, as the Box plots in Fig. 1 demonstrate. In 2013–14, the median prevalence rate in communities without LAF remained almost unchanged compared to 2011–12 (11.0; *p* = 0.94). In communities with LAF it rose slightly to 5.6 in 2013–14 (*p* = 0.93) – still around half the rate in communities without LAF (*p* = 0.37). Table 3 shows the median rates, inter-quartile ranges and ranges for communities in 2011–12 and 2013–14.

**Table 3:** Sniffer prevalence rate per 1000 population (aged 5–39 years) by LAF availability in 2011–12 and 2013–14 (41 communities)

Year	Number of communities	Total sniffers	Population 5–39 years	Sniffer prevalence rate/1000 pop		
				Median	Inter-quartile range	Range (Min-Max)
2012						
No LAF	11	111	3946	11.2	0–38.9	0–140.2
LAF	30	178	12,061	3.9	0–13.4	0–186.6
2014						
No LAF	10	39	2517	11.0	5.7–18.7	0–52.6
LAF	31	165	13,490	5.6	0–18.8	0–87.9

**Fig. 2:**
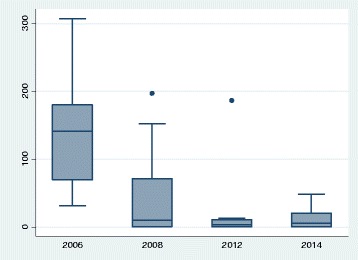
Petrol-sniffing prevalence per 1000 population in communities with LAF (15 communities)

Among the 17 communities for which longer term data are available, two had no LAF during the entire period. In one of these communities there were 43 sniffers in 2005–06, 22 in 2007–08, 5 in 2011–12 and 2 in 2013–14, in the other there were no sniffers in 2005–06, 2007–08 and 2011–12 and 10 in 2013–14. These numbers do not allow for any interpretation of trends. The remaining 15 communities had LAF over the entire eight-year period. In these, the median rate of petrol sniffers declined by 96%, from 141.6 per 1000 population in 2005–06 to 5.5 in 2013–14 (*p* < 0.05). While there was a slight increase in the median rate of sniffers between 2011–12 and 2013–14 from 3.7 to 5.5, this was not statistically significant (*p* = 0.69). Table 4 shows the prevalence rates and ranges, while Fig. 2 shows the box plots of prevalence rates at each of the four time points.

**Table 4:** Sniffer prevalence rate per 1000 population (aged 5–39 years) by year in communities with LAF (15 communities)

Year	Total sniffers	Sniffer prevalence rate per 1000 population		
		Median	Inter-quartile range	Range (Min-Max)
2006	604	141.6	70.1–180.0	31.4–307.3
2008	265	10.4	0–71.4	0–196.9
2012	93	3.7	0–11.1	0–186.6
2014	66	5.5	0–20.8	0–48.5

The 15 communities in this sub-sample were located in the Northern Territory, South Australia, Western Australia and the far northern region of Queensland. While the trajectory of sniffing frequencies was not the same across the regions in which the communities were located, all of the regions experienced a decline in sniffing numbers over the period under review.

In most communities where LAF had been introduced, it was described as having had a beneficial impact (26 out of 31 communities in 2013–14, or 83.9%) and as being widely accepted. In one, community leaders described LAF as having ‘saved’ their own and nearby communities from an onslaught of petrol sniffing. In several communities, LAF was described as having become so embedded in the community that many younger people were probably not aware that it was any different from RULP, or that the community was even using LAF. Even in these communities, however, leaders cautioned that, if ever RULP were to return, it would take little to rekindle old practices of sniffing.

Support for LAF, however, was not unqualified. In a little over half of the survey communities (21 communities), fieldworkers were told about the damage that LAF was believed to inflict on engines, especially outboard motors and other small engines such as motorbikes, lawn-mowers and whipper-snippers. In one community, these beliefs had gained enough currency to result in the community electing to suspend use of LAF. On the other hand, several people interviewed argued that mechanical problems attributed to LAF were in fact due to other causes, such as dirt getting into engines or rust in old petrol storage tanks. Even among those who believed that LAF was harmful to engines, some supported its use. One woman, who claimed to have had to replace her fuel pump three times because of LAF, remarked: ‘it’s just a car; we’re talking about lives’. She had seen the effects of petrol sniffing on two of her own uncles who, as she put it, now ‘walk wobbly’.

This study was not designed to gather systematic data on drug use other than petrol sniffing. However, fieldworkers were instructed to collect qualitative data on use of other drugs, including other inhalants, and on evidence of substitution of other drugs for RULP. The results of their inquiries are necessarily anecdotal; nonetheless, they serve a useful purpose in locating petrol sniffing within a context often driven by opportunistic use of a variety of substances.

The most widely used volatile substance, as one would expect, was RULP, with reports of its use in 24 communities. This was followed in popularity by deodorants (17 communities), glue (9 communities) and aerosol paints (9 communities). Several other inhalants were also mentioned between one and three times. These included cooking gas, fly spray, hair spray, lighter fluid, nail polish, and premium unleaded petrol. (It is also possible that some of the petrol sniffed and identified as RULP may in fact have been premium). There were also two reports of would-be users adding polystyrene to LAF in the (unfulfilled) hope of becoming intoxicated.

In the months following completion of fieldwork for this project, public allegations were made regarding burgeoning use of ‘ice’, or crystal methamphetamines, in Indigenous communities [30–32]. In four communities in this study, fieldworkers were told anecdotally that ice may have been present in the community, but in no community did fieldworkers encounter any first hand evidence of ice or other illicit drugs.

Of much greater concern than these behaviors in many communities were the high levels of concern regarding alcohol and cannabis. For analytical purposes, interviewees’ qualitative assessments regarding the impact of both of these substances were coded in one of three categories: (a) substance reported as not present in the community, (b) substance reported as present, but not a cause for concern, (c) use of substance reported to be heavy and/or a major concern. In the case of cannabis, a fourth category was also created, to cover communities in which fieldworkers reported conflicting assessments of the presence and/or severity of associated cannabis-related problems. In around two-thirds of the 41 communities (27, or 65.9%), cannabis use was reported as being heavy and/or a major cause for concern. In just over half of the communities (21, or 51.2%), alcohol use was described as heavy and/or a major cause for concern. In 14 communities (34.1%), fieldworkers reported heavy use and/or major concerns with both alcohol *and* cannabis. Alcohol was implicated by interviewees in binge drinking, grog running, family violence, injury and deaths, while cannabis was linked to drug-induced psychoses, fighting and property damage. As one person’s remarks attested, cannabis can cause problems even in its absence:

*Young people hassle old people to get money for gunja [a slang term for cannabis] and they steal baskets to sell. Young people get cranky with older family members when there’s no gunja. I was told by an old lady recently that she was pushed over by young people wanting food and money.*

The decline in petrol sniffing, together with evidence of high levels of alcohol and cannabis use.point to a possibility that some petrol sniffers may have substituted other substances for petrol. In 14 communities (34.1%), fieldworkers were told that the decline in petrol sniffing appeared to have led to an increase in use of cannabis, alcohol, or other drugs more generally. Few observers, however, drew a direct causal link. In many communities, the growth in cannabis use preceded the decline in petrol sniffing, and was generally attributed to a number of interconnected factors, including its ready availability, its social acceptability in the community, and the boredom of many young people. In six communities, informants told fieldworkers that, in general, alcohol, cannabis, inhalants including petrol and, sometimes, other drugs, were all regarded as interchangeable; if you could not access one, you would find another.

As a young person in one community put it:

*All the kids I sniffed with stopped but they are smoking gunja now… When I left petrol I was smoking gunja, when I left gunja I was drinking.*

To compare levels of concern with alcohol and cannabis in the 18 communities that recorded a decline in petrol sniffing between 2011–12 and 2013–14 with levels of concern in those communities that recorded no decline, levels of concern were dichotomized, with ‘heavy use and/or major concerns’ as one category, and ‘not present, present but not a concern, or conflicting reports’ as the other category. The results are shown in Table 5.

**Table 5:** Reported concern with cannabis and alcohol in 2013–14 and change in petrol sniffing prevalence from 2011–12 to 2013–14

Reports of concern by substance	Change in petrol sniffing prevalence between 2011–12 and 2013–14		Total
	Decline (%)	No change/increase (%)	
Alcohol			
Heavy use and/or major concern	6 (33.3)	15 (65.2)	21 (51.2)
Not present or present, but not an issue	12 (66.7)	8 (34.8)	20 (48.8)
Total	18 (100.0)	23(100.0)	41 (100.0)
Cannabis			
Heavy use and/or major concern	12 (66.7)	15 (65.2)	27 (65.9)
Not present or present but not an issue or conflicting reports	6 (33.3)	8 (34.8)	14 (34.1)
Total	18 (100.0)	23(100.0)	41 (100.0)
Other inhalants			
Currently used	10 (55.6)	13 (56.5)	23 (56.1)
No evidence of current use	8 (44.4)	10 (43.5)	18 (43.9)
Total	18 (100.0)	23(100.0)	41 (100.0)

Table 5 shows that communities reporting a decline in prevalence of petrol sniffing between 2011–12 and 2013–14 were no more or less likely than other communities in the sample to report heavy use and/or major concerns with cannabis use. In both groups of communities, around two-thirds reported major concerns (RR 1.02, 95% CI 0.66–1.59, *p* = 1.00). Similarly there was no association between communities reporting use of other inhalants in 2013–2014 and a decline in petrol sniffing between 2011–12 and 2013–14 (RR 0.98, 95% CI 0.57–1.70, *p* = 1.00). In the case of alcohol problems, however, there was a strong association, though possibly not in the direction that might have been anticipated. Far from a decline in petrol sniffing being associated with an increase in alcohol problems, communities reporting a decline in petrol sniffing between the two survey times were *less* likely than other communities to report heavy alcohol use and/or major concerns with alcohol problems (RR 0.51, 95% CI 0.25–1.05, *p* = 0.06).

Efforts to deal with petrol sniffing and other drug-related problems in remote Indigenous communities have long been hampered by inadequate intervention services, inadequately resourced youth and recreation programs, and a dearth of training and employment opportunities. Our study found evidence of improvements in access to intervention services. Fieldworkers were asked to document the availability or otherwise of personnel with either mental health or AOD (alcohol and other drug) qualifications. In our 2007–08 study of 20 communities, 30% of communities had access either to a regular visiting service or an on-site service. By 2013–14, the proportion had risen to just over half (51.3%) of the 41 communities surveyed.

Youth, sport and other recreational activities in communities ranged from being virtually non-existent (four communities) to apparently adequately funded and satisfactory (nine communities), with the majority (26 communities) falling in between these two extremes – that is, activities were in place, but the programs struggled in the face of problems of funding, staffing and/or facilities. (In two communities, insufficient information was collected to allow for any assessment of the programs.)

Functioning programs created their own energy, as the following report demonstrates:

*The Shire now has a manager of the Youth Program on the Lands and there are two experienced youth workers in the community running the local program. There are activities every day after school times utilising the basketball court, rec hall, drop-in centre, gym, oval and pool (in summer). There are movie nights and discos a couple of evenings a week.*

Unfortunately, the following field report describes a more typical scenario than the one above. It comes from a community of about 350 people located a little over 300 km from Alice Springs:

*There is no dedicated youth centre building. The basketball court is the centre of activity. The toilets don’t work properly. The youth worker has renovated an air-conditioned shipping container as an art and craft space for little kids. The kitchen is currently unusable and he cooks at home. He is trying to renovate the complex so that at the end of the year it is in a fit condition for his next year or the next person. He is very tired, working 70-hour weeks and has no phone or internet at home. He feels very supported by CAYLUS [Central Australian Youth Link-up Service, based in Alice Springs] but feels that the Shire is very unsupportive.*

Similarly, in a few communities employment and training activities appeared to be opening up genuine opportunities for young people. Here is a fieldworker’s report from a community in Far North Queensland:

*There was a continued sense of things happening - there were locals employed in various roles in the agencies and the shop. [One program] had 325 on the books and 47 in jobs aged 16-60 yrs. Training run in last 12 months included Cert 2 in Tourism, Cert 2 in Indigenous Housing and Cert 2 in Hospitality.*

*There was a particular emphasis on a group of young people aged 16-25, and 13 had travelled to Tully for leadership training – ten of these were regular attenders and there were plans that included work with elders recording stories and catering, repairing a damaged park, a youth newsletter, development camps and designing their own uniform. It is planned that this group will be the core of the Youth Hub. The program is now more compliance-based and if people don’t work they don’t get paid. Most people have to do 20 hours per week with some up to 30.*

More typical, unfortunately, was the situation described in another community:

*There are not many job opportunities at [name of community]. RASAC (Regional Anangu Service Aboriginal Corporation) employs half a dozen community members and the store, clinic, school, aged care, Catholic Care, Money Mob, the internet centre and the Well Being Centre also employ people. Skill Hire, the RJCP provider, has 150 people on their books. They cannot provide work for everyone for 20hrs/week so they give them activities - women have been picking up tyres from the tip, painting them and using them as garden beds, the men have recently completed landscaping at the school and now are cleaning up the yards. Skill Hire organizes training for workers in connection with the activities they undertake. As one of the Skill Hire coordinators put it ‘they get trained to death - they have bobcat certificates, excavator tickets - but nowhere to use them’.*

With exceptions in a few communities, attempts to generate employment opportunities for young people were found to be plagued by one or more of the following problems:

- dearth of training programs;
- dearth of employment opportunities;
- where employment opportunities are in principle available – for example, in nearby mines – disqualification of otherwise eligible young people because of issues such as prior drug offences, current drug use, or licence cancellations;
- absence of basic literacy and numeracy skills; and
- lack of motivation on the part of young people.

Each one of the above points raises issues that reach beyond the scope of this paper or our inquiry. It is important to note, however, that this is the context within which attempts to address alcohol and other drug problems, including petrol sniffing and the rollout of LAF, are played out.

## Discussion

The study demonstrates that the introduction of LAF has been accompanied by a significant decline in the prevalence of petrol sniffing. In 15 communities for which data is available over an extended period from 2005–06 to 2013–14, and which stocked LAF during the entire period, the median prevalence rate of petrol sniffers declined from 141.6 per 1000 population to 5.5. Among the 41 study communities in the recent study, communities with LAF had prevalence rates lower than communities without LAF (5.6 compared with 11.0 in 2013–14). Although the differences were not statistically significant, this is an outcome of small absolute numbers of sniffers in communities and the small number of communities, leading to high variability.

It is not possible, on the basis of these findings, to ascribe the decline in petrol sniffing entirely to the introduction of LAF. The rollout of LAF was not conceived as a trial or research exercise, but rather as a policy initiative under which LAF has been made available to communities as and when they request it and demonstrate a need. Consequently, there are no control communities with which to compare prevalence. Among the 17 communities for which long term data are available, in only two of them was LAF not available – hardly a basis for comparison. Among the 41 communities in the more recent study, as indicated above, 28 communities had LAF at the time of both data collections, eight had no LAF at either time, and the remaining five communities changed their LAF status during the course of the study. Comparison of those having LAF with those not having it, as shown above, suggests that LAF has reduced prevalence of sniffing, but other contextual differences may well have contributed to the observed outcomes.

The quantitative evidence of a decline is corroborated by the testimony of community residents and service providers, among whom there is a widely shared belief that LAF has been a major factor in reducing petrol sniffing. Similar observations were made by several witnesses to a 2012 Senate inquiry into legislative options for supporting the rollout of LAF [24]. Support for LAF in communities was qualified by a widespread belief that LAF was harmful to engines, especially small engines. Results of independent tests suggest that the allegations are unfounded. In 2007 the Royal Automobile Association of South Australia reported on investigations conducted among mechanical repairers, services stations and automobile parts suppliers in Alice Springs, in which mechanical symptoms that had been attributed to Opal LAF were examined. The report concluded that all of the problems associated with defective fuel pumps, fuel hoses and fuel injection systems were due to normal ageing under the prevailing central Australian conditions, and that there was no evidence of Opal LAF harming vehicle engines, generator sets or outboard motors [33]. They did, however, report that, because Opal requires slightly more oxygen than RULP, minor tuning adjustments might sometimes be necessary to maintain the idling quality of some older vehicles with carburettors. In another study, also published in 2007, a laboratory analysis of four samples of Opal LAF drawn from bowsers in Alice Springs and Yulara in January 2007 found that all samples complied with the National Standard set out in the *Fuel Standard (Petrol) Determination 2001*. The analysts reported that, in their opinion, the fuel tested would have no adverse environmental or engine operating effects; on the contrary, the low content of sulphur, benzene, other aromatics and olefin meant that use of the fuel would entail an environmental and health benefit. It would also, in their view, enhance engine efficiency [34].

From a policy point of view, the persistence of beliefs about adverse effects of LAF on engines, despite evidence to the contrary, and despite considerable efforts already made by government and other agencies to dispel misconceptions, poses an ongoing challenge that cannot be addressed simply by pointing to the objective evidence. Shared beliefs have consequences regardless of their objective validity. Our study found one instance of a community that actually stopped using LAF because some people in the community believed that it was damaging outboard motors. The question of *how* best to counter misconceptions requires ongoing attention.

The study found evidence of a belief in some communities that reductions in petrol sniffing may have led to substitution with other drugs, including other inhalants and cannabis. Given that much drug use by young people – in remote communities as elsewhere – takes the form of opportunistic polydrug use, this is not surprising. The findings, however, do not support a simple cause-and-effect interpretation. Changes in drug use patterns are normally a product of many causal factors. In the case of Indigenous Australian communities, the apparent widespread, heavy consumption of cannabis that has given rise to the concerns reported in this study predates the introduction of LAF. As several studies have reported, the availability and consumption of cannabis in remote Indigenous communities increased dramatically from the mid-1990s, driven by rising demand and highly organised profiteering mainly by sources outside the communities themselves [35, 36]. Moreover, this study found that communities in which the prevalence of petrol sniffing declined between 2011–12 and 2013–14 were no more or less likely than other communities to report heavy cannabis use or cannabis-related problems in 2013–14.

Communities reporting a decline in petrol sniffing were, however, *less* likely than other communities to report heavy alcohol use and/or a major concern with alcohol problems. It is not immediately clear why this should be so, and the question warrants further examination. While decisions on whether or not to sniff petrol, drink alcohol or use other drugs are taken by individuals, the mechanisms available for managing alcohol and other drug use in local communities operate through families, communities and states/territories, as well as individuals. Levels of petrol sniffing, alcohol-related problems and other drug use may, to some extent at least, be different aspects of a single attribute which can be tentatively conceptualized as ‘community capacity’. A useful extension of the findings of this study would be to examine the relationship between petrol sniffing, other drug use, and community capacity.

At the same time, caution is needed in interpreting these results. While the reported levels of petrol sniffing are quantitative estimates, the data on cannabis, alcohol and other drugs are all, as indicated earlier, qualitative and anecdotal.

The improvements in both the quantity and quality of alcohol and other drug services in communities reported in this study, and the limited improvements in provision of youth and recreation services, are to be welcomed. However, the findings also point to a continuing pattern of unstable and often inadequate funding and other resource problems in these programs, and the need for ongoing improvements.

The main limitation of the study is the absence of an adequate sample of control communities. Among the 17 communities for which long term data are available, only two did not have LAF throughout the period under review. Among the larger sample of 41 communities, the eight communities that did not have LAF at either data-collection time furnish a basis for comparison, but do not constitute a matched group of control communities. A second limitation is the absence of population projections for two data collection times – 2007-08 and 2013–14. As a result, it was necessary to base population estimates for these years on census returns for 2006 and 2011 respectively. A third limitation is our reliance on proxy respondents for estimates of petrol sniffing prevalence. Finally, interpretation of statistical findings was constrained by small numbers.

## Conclusions

The support for the use of LAF in remote Indigenous communities as a deterrent to petrol sniffing by successive Australian governments is an example of an adequately funded program that has enjoyed broad political support, and consistency in application. Insofar as governmental decisions to support the provision of LAF to individual communities are taken in response to requests from those communities, it is also an example of government policy complementing community initiatives. The findings from this and earlier studies of the LAF rollout demonstrate the benefits to be derived from this approach to policy-making. LAF is not a ‘magic bullet’ for eradicating petrol sniffing. Further research is needed to identify the conditions under which LAF is and is not effective in reducing petrol sniffing. From thepoint of view of policy and programs, it is vital that the continuing need for other programs and services, also demonstrated in this study, be addressed if the gains in reducing petrol sniffing are to be consolidated.

## Abbreviations

AOD: alcohol and other drugs
APY: Anangu Pitjantjatjara Yankunytjatjara
CAYLUS: Central Australian Youth Link Up Service
LAF: Low Aromatic Fuel
RCT: Randomised Control Trial
RULP: Regular Unleaded Petrol
VSM: Volatile Substance Misuse

## Footnotes

1 Neither BP nor Viva has released full details of the composition of its fuels, presumably for commercial reasons. Both fuels have a Research Octane Number (RON) of 91. In both, the proportion of aromatic hydrocarbons, including toluene and benzene, is no more than 5%, compared with up to 25% in RULP. For further technical details, see: http://www.msds.bp.com.au/msds.aspx?msdsno=0000003434 and http://www.shell.com.au/motorists/shell-fuels/msds-tds/_jcr_content/par/textimage_278c.stream/1468572330831/1d58662064a9eb79c9f108f3ee08a6244fe6fced69a8e4e1012685ae7c76f04c/tds-shell-unleaded-petrol-low-aromatic.pdf.

2 Australian Government Department of Prime Minister and Cabinet, *Pers. Comm.*

3 Because the reports for both of these studies listed identifiable Indigenous communities, they were not published. However, summary reports containing de-identified data were subsequently posted online, and are currently on the Department of Prime Minister & Cabinet website at https://www.dpmc.gov.au/resource-centre/indigenous-affairs/data-collection-petrol-sniffing-prevention-program-report-summary and https://www.dpmc.gov.au/resource-centre/indigenous-affairs/evaluation-impact-opal-fuel-executive-summary. They are also reproduced as appendices in d’Abbs and Shaw 2016.
